# Use of Twitter Among Local Health Departments: An Analysis of Information Sharing, Engagement, and Action

**DOI:** 10.2196/jmir.2775

**Published:** 2013-08-19

**Authors:** Brad L Neiger, Rosemary Thackeray, Scott H Burton, Callie R Thackeray, Jennifer H Reese

**Affiliations:** ^1^Brigham Young UniversityDepartment of Health ScienceProvo, UTUnited States; ^2^Brigham Young UniversityDepartment of Computer ScienceProvo, UTUnited States

**Keywords:** Twitter, social media, engagement, Internet, audience

## Abstract

**Background:**

Social media offers unprecedented opportunities for public health to engage audiences in conversations and collaboration that could potentially lead to improved health conditions. While there is some evidence that local health departments (LHDs) are using social media and Twitter in particular, little is known about how Twitter is used by LHDs and how they use it to engage followers versus disseminating one-way information.

**Objective:**

To examine how LHDs use Twitter to share information, engage with followers, and promote action, as well as to discover differences in Twitter use among LHDs by size of population served.

**Methods:**

The Twitter accounts for 210 LHDs were stratified into three groups based on size of population served (n*=*69 for less than 100,000; n*=*89 for 100,000-499,999; n*=*52 for 500,000 or greater). A sample of 1000 tweets was obtained for each stratum and coded as being either about the organization or about personal-health topics. Subcategories for organization included information, engagement, and action. Subcategories for personal health included information and action.

**Results:**

Of all LHD tweets (n=3000), 56.1% (1682/3000) related to personal health compared with 39.5% (1186/3000) that were about the organization. Of the personal-health tweets, 58.5% (984/1682) involved factual information and 41.4% (697/1682) encouraged action. Of the organization-related tweets, 51.9% (615/1186) represented one-way communication about the organization and its events and services, 35.0% (416/1186) tried to engage followers in conversation, and 13.3% (158/1186) encouraged action to benefit the organization (eg, attend events). Compared with large LHDs, small LHDs were more likely to post tweets about their organization (Cramer’s V=0.06) but were less likely to acknowledge events and accomplishments of other organizations (χ^2^=12.83, *P*=.02, Cramer’s V=0.18). Small LHDs were also less likely to post personal health-related tweets (Cramer’s V=0.08) and were less likely to post tweets containing suggestions to take action to modify their lifestyle. While large LHDs were more likely to post engagement-related tweets about the organization (Cramer’s V=0.12), they were less likely to ask followers to take action that would benefit the organization (χ^2^=7.59, *P*=.02. Cramer’s V=0.08). While certain associations were statistically significant, the Cramer’s V statistic revealed weak associations.

**Conclusions:**

Twitter is being adopted by LHDs, but its primary use involves one-way communication on personal-health topics as well as organization-related information. There is also evidence that LHDs are starting to use Twitter to engage their audiences in conversations. As public health transitions to more dialogic conversation and engagement, Twitter’s potential to help form partnerships with audiences and involve them as program participants may lead to action for improved health.

## Introduction

Organizational use of social media is expanding in both the private and public sectors [1,2]. Some evidence suggests social media is also being adopted in public health and health education settings [3-5]. Based on preliminary studies, inferences about the use of social media in public health settings include the following: (1) it is in an early adoption stage, (2) it tends to be used more often in urban and high-density populations compared with rural communities, and (3) it is used primarily to share information through one-way communication (ie, one sender and one or more receivers as with traditional mass media).

Some evidence suggests one-way communication delivered through social media can play an important role during disease outbreaks [6] and emergency response and relief efforts [7-9]. One-way communication can also create a foundation for more complex functions such as dialogue and mobilization [10]. However, limiting social media use to one-way communication decreases its interactive capacity to engage audiences. In fact, engaging audiences in two-way, dialogic, or conversational communication is the central purpose of social media [11]. So, while social media can be used to disseminate health information, it should also be used to create dialogue and engage audiences.

Engagement is a key element in mobilizing and building communities and the benefit of social media is not maximized unless it engages members of the community [10]. In the context of health promotion and social media, engagement has been defined as connections between people that contribute to a common good [12] and result in some type of action on behalf of the individual or organization [13]. This implies a mutual awareness and interaction between public health organizations and their audiences that lead to mutually beneficial outcomes. It has been recommended that social media be used more strategically within public health settings to engage audiences in ways that lead to action for health, such as involving them in the creation or delivery of programs or recruiting them as participants or recipients of programs, services, and activities [13].

One social media option for public health to engage audiences more effectively is Twitter, an information network composed of 140-character messages [14]. From 2010 to 2012, daily Twitter use experienced a four-fold increase; currently 15% of online adults use Twitter [15]. Corporate use of Twitter is also increasing significantly [16] and is used to enhance brands, increase visibility, support customers, network, communicate internally, generate leads, and support other online presences [17]. Twitter has re-launched its Twitter for Business site as an internal service for businesses who want to use Twitter to build communities and market and promote their products [18].

Nonprofit organizations are also are using Twitter in a number of ways to promote their organizations and mobilize their audiences. In a study conducted among the 100 largest nonprofit organizations in the United States, Lovejoy and Saxton [10] examined the use of Twitter to determine the types of tweets (ie, messages) these organizations were sending to their audiences. Their message classification approach included three categories: (1) information, or tweets about the organization (eg, highlights from events, news, facts, reports, etc), (2) community, which involved tweets that promoted dialogue and facilitated the creation of online communities, and (3) action, that involved tweets aimed at getting followers to do something for the organization. The researchers reported that 59% of tweets were informational in nature, 26% related to community building, and 15% related to action. They concluded that social media holds the potential to create opportunities for interpersonal engagement that are qualitatively different than traditional communication approaches and that to date, the nonprofit sector has overutilized the information function of social media and underutilized its interactive and dialogic components.

Research conducted among state health departments suggests Twitter is the most commonly used social media application in public health [3,4]. Initial analysis of county or local health departments (LHDs) revealed they are also using Twitter. According to the National Association of County and City Health Officials (NACCHO) [19], there are 2565 LHDs in the United States representing the largest delivery arm of public health services in the country.

LHD jurisdictions are classified as county (68%), multicounty (8%), and city (21%) with the remainder categorized as “other”, including LHDs serving multiple cities and LHDs serving a county and a city not lying within the county boundaries [19]. However, LHD jurisdictions do not necessarily correlate with size of population served. For example, some county health departments serve small populations and others serve large populations and so forth. LHDs are granted legislative authority through codes and statutes and can be governed by local authorities (eg, local board of health or county or city elected officials) or by a state health agency or both [19].

The population-based primary prevention services provided most often by LHDs relate to chronic diseases and associated determinants. Clinical services provided most often by LHDs involve adult and child immunizations, infectious disease surveillance and screenings, food service inspections, and environmental health surveillance [19].

The LHD workforce constitutes a broad range of public health subdisciplines and professionals (eg, physicians, nurses, health educators, environmental health workers, emergency preparedness staff, nutritionists, etc) and thereby provides a potentially rich sample of Twitter use within public health [19]. However, to date, there is a paucity of literature about LHD use of Twitter, though one recent study did report how LHDs are using it to disseminate diabetes-related information [20]. Furthermore, while initial studies have reported frequency distributions of social media applications used in public health settings, no studies reported to date have investigated how social media is used within public health to engage audiences and involve them in actions related to programs and services.

Therefore, the purpose of this study was to examine how LHDs were using Twitter to communicate with and engage audiences. The following research questions guided this study:

1. Are LHDs more likely to use Twitter to share information, to engage with followers, or to promote action among followers?
2. Are there differences between LHDs serving varying population strata (eg, small, medium, and large) in the types of Twitter messages they post?
3. What health topics are LHDs tweeting about?

## Methods

### Sample

A list of all LHDs in the United States was obtained from the NACCHO (n=2565). Presence of a Twitter account was determined by three means. First, researchers visited the homepage of each LHD based on the website address provided by NACCHO. Researchers documented the presence or absence of a social media button indicating the LHD had a Twitter account. If there was no visual sign of Twitter on the homepage, a Google search was performed. Finally, on the Twitter homepage, each LHD name was entered in the search bar to confirm the presence of the account.

To be included in the study, the LHD had to have a Twitter account (n=306) and have posted a minimum of 50 tweets (n=210). Researchers then divided this list into three stratum: small, medium, and large based on size of population served (less than 100,000; 100-499,999; 500,000), hereafter referred to as small, medium, and large LHDs. Initial analysis showed a statistically significant difference in the total number of tweets a LHD posted based on size of population served, thereby confirming the need to stratify the study population. In the final sample, there were 69 small (32.8%), 89 medium (42.4%), and 52 large (24.8%) LHDs.

A complete list of tweets made by each LHD account was obtained using the Twitter Application Programming Interface (API) during July 2012. Because the Twitter API limits the maximum number of tweets that can be retrieved to the most recent 3200 per account, this limit was considered a complete tweet list for any LHD account that exceeded 3200 tweets. A total of 1000 tweets were sampled from each stratum for a total of 3000 tweets. To determine the number of tweets to be sampled from each LHD in the stratum, researchers divided 1000 by the number of LHDs in that stratum. There was an average of 14.5 tweets per LHD in the small stratum, 11.2 per LHD in the medium, and 19.2 tweets per LHD in the large stratum. Researchers randomly sampled tweets using a uniform distribution from each LHD account’s list. This methodology was selected to reduce bias, including overrepresentation of tweet frequency during a specific event (eg, National Public Health Week), or underrepresentation due to irregularity of Twitter posts, or the LHD recently establishing a Twitter account.

### Instrumentation

The researchers coded tweets based on the methodology used by Lovejoy and Saxton [10], though modified to reflect public health practice. The coding was designed to determine the purpose of the tweet including whether the LHD was using Twitter to disseminate information, foster a sense of community or engagement with the community, or motivate audiences to action. Information sharing was defined as “one-way interaction, the exchange of information from the organization to the public” (page 343) [10]. Action was defined as “messages that aim to get followers to do something for the organization” (page 345) [10] or for their personal health. Based on social media terminology relevant in public health literature, the term “engagement” was used in lieu of “community” [13,21]. However, the researchers retained Lovejoy’s and Saxton’s definition of community for engagement, which was using Twitter to “interact, share, and converse with stakeholders in a way that ultimately facilitates the creation of an online community with its followers” (page 343) [10].

Tweets were first coded as being either about the organization or about personal-health topics. Then each tweet was coded for each of the categories described above (information, engagement, or action). Each classification category was mutually exclusive. Organization information included topics such as events or services provided, news, facts, reports, or job announcements. Organization engagement tweets were conversational in nature and gave thanks and recognition for doing something for the organization, acknowledged other organization’s events, responded to public reply messages, asked for a response to a Twitter post, asked for feedback or suggestions, asked people to follow them or become a fan, or asked followers to spread the word or retweet the Twitter post. Action-based organization tweets invited followers to attend events, attend meetings and provide input, complete a survey, donate goods or money, volunteer time, or participate in lobbying or advocacy.

Personal-health tweets were limited to information and action. Information-related tweets involved general public health information (eg, eating foods rich in folic acid or taking a prenatal vitamin before you are pregnant can help prevent birth defects), risk communication related to disease outbreaks or natural or manmade disasters, or reports about public health conditions (eg, new report about obesity in America). Personal-health action-based tweets included messages to receive preventive health screenings (eg, get a mammogram), modify one’s lifestyle (eg, make sure to walk for 30 minutes today), or to learn more and increase knowledge (eg, Now is the time to prepare for a disaster! Learn what you can do.). Each classification category was mutually exclusive.

The degree to which a tweet was considered interactive and attempted to engage the audience was determined by the presence of three components: (1) an @ reply symbol, signifying that the LHD was responding to a post made by another Twitter follower, (2) @username, indicating that the LHD was directing its post to a specific user, and (3) the use of personal pronouns [22,23]. The level of sophistication of each tweet was noted by (1) whether it was a truncated tweet, meaning that the LHD posted something on one platform (eg, Facebook) that was then posted to the Twitter account, (2) if it was a retweet, and (3) the existence of hashtags within the tweet. Truncated tweets and retweets denote that the LHD is not developing content specifically for Twitter but sharing what others have posted. Hashtags, which are used to categorize tweets so users can easily follow topics posted on Twitter, are also reflective of a more advanced Twitter user. Tweets were also coded as to whether the follower was redirected to another source for more information. This signified that the LHD was using Twitter as a one-way communication tool and was linking people to more information.

Two research assistants pretested the coding instrument. Ten tweets from ten LHDs were selected for inclusion in the pretest analysis. Based on the results, minor adjustments were made to the coding sheet to clarify definitions. In some instances, the tweet content could be coded for more than one category. However, following Lovejoy and Saxton’s methodology [10], research assistants identified the primary purpose of the tweet and coded it accordingly. If a personal-health tweet could be categorized as either information or action, it was coded as action with the assumption that taking action was the focus of the tweet. If a tweet included only a URL link with no other text, the tweet was viewed as only a redirect and was not coded further.

Four research assistants hand-coded all tweets. Two pairs of research assistants each coded half the tweets and then compared answers and resolved discrepancies. If there was a discrepancy between the two sets of coded data, the pair discussed each issue until consensus was reached. Discrepancies most often occurred because of a simple error related to data entry or a misinterpretation of the tweet.

### Data Analysis

The chi-square test was used to assess differences between small, medium, and large LHDs. Standardized residuals were analyzed to determine which cells contributed significantly to the results. When a standardized residual is greater than 2, the cell is contributing significantly to the differences between groups [24]. The Cramer’s V statistic was used to test the strength of association between two categorical variables. This is an appropriate test to use after the chi-square statistic is found to be significant [25].

## Results

In the final sample, there were 69 small, 89 medium, and 52 large LHDs for a total of 210 LHD Twitter accounts. LHDs across all three strata (large, medium, and small) had a mean of 526.8 followers (ie, another Twitter user following the LHD) (range 9833, SD 1112.4) and followed a mean of 156.3 other Twitter users (range 4237, SD 342.99). Although large LHDs had more followers, followed more users, and posted more tweets compared with medium or small LHDs, these differences were not statistically significant as determined by the chi-square statistic (Table 1). The earliest Twitter account was established in June 2008 (Sacramento County, CA Public Health), and the most recent account was created in April 2012 (Independence, MO Health Department).

The majority of tweets (85.9%, n=2578) were original posts by the LHD. However, 15.2% (455/3000) of tweets were truncated, meaning the tweet originated from another source and was not fully displayed on Twitter. Just over 7% (7.3%, 218/3000) of posts were directly from Facebook. Almost 20% (19.7%, 592/3000) of tweets included the @ symbol, though this was an @reply only 1% (n=30) of the time (ie, the LHD was responding to another Twitter user’s post). Hashtags, used to mark keywords or topics in a tweet were used 16.2% (486/3000) of the time. Almost three-fourths of tweets (73.7%, 2211/3000) directed users to another source for more information through a website URL link (eg, the Centers for Disease Control and Prevention site). One third of tweets (36.7%, 1102/3000) used personal pronouns.

Overall, LHD tweets more often related to personal health (56.1%, 1682/3000) compared with information about the organization (39.5%, 1186/3000). There were a small number of posts that were non-health related and included topics such as news, events, and community happenings (2.2%, 65/3000).

### Personal-Health Tweets

Of the personal-health tweets, the majority involved factual information (58.5%, 984/1682). Less than half of the personal-health tweets (41.4%, 697/1682) included an imperative verb that encouraged people to take action for their own health benefit. Of those tweets that included an action, 46.8% (326/697) related to knowledge (eg, learn more here), 37.2% (259/697) related to lifestyle behavior modification (eg, get more physical activity), and 10.4% (72/692) encouraged people to get preventive health screenings (eg, go for a mammogram).

### Organization-Related Tweets

Organization-related tweets primarily represented one-way information about the organization (51.9%, 615/1186). The majority of these tweets (65.5%, 403/615) focused on events or services that the organization provided such as a flu clinic or breast-feeding class. Only 13% (13.3%, 158/1186) of organization-related tweets encouraged people to take action to benefit the organization. Of tweets aimed at getting people to take action, asking people to attend events was most common (69%, 109/158).

Just over a third (35%, 415/1186) of organization-related tweets were trying to engage their audiences in conversation. The most common way to engage with audiences was through acknowledgement of other organizations’ events (23.9%, 99/415) and giving thanks for recognition (17%, 70/415). In addition, several tweets appeared conversational in nature (24.1%, 100/415), while not fitting the exact engagement categories identified in the coding sheet (eg, “Back from a refreshing but hot walk. Now following heat safety guidelines and drinking some water #heatwave”).

### Tweets by Size of Population Served

There were significant, yet weak associations between size of population served by the LHD and several of the study variables (Table 2). Analysis of standardized residuals showed that either the large or small LHDs were contributing to the significant chi-square value, but never the medium-sized LHDs. Small LHDs were more likely to have truncated tweets (Cramer’s V=0.18) and use personal pronouns (Cramer’s V=0.06). Large LHDs were more likely to use the @ symbol in their original tweets (Cramer’s V=0.09), redirect followers to a different website or link (Cramer’s V=0.096), and use hashtags (Cramer’s V=0.14)

There was a significant association between LHD size and posting personal-health–related tweets. Examination of standardized residuals showed that small LHDs were less likely to do so (Cramer’s V=0.08). For personal-health–related tweets, small LHDs were less likely to post tweets containing suggestions to take action to modify their lifestyle (χ^2^=8.90, *P*=.01, Cramer’s V=0.11).

Overall, small LHDs were more likely to post tweets about the organization (Cramer’s V=0.06), and large LHDs were more likely to post organization-engagement–related tweets (Cramer’s V=0.12). For organization-engagement tweets, small LHDs were least likely to acknowledge events and accomplishments of other organizations (χ^2^=12.83, *P*=.02, Cramer’s V=0.18). Large LHDs were also less likely to ask followers to take action that would benefit the organization (χ^2^=7.59, *P*=.02, Cramer’s V=0.08).

**Table 1:** Number of tweets, followers, and who they follow by local health department size.

	Small n=69	Medium n=89	Large n=52	Total n=210	*P* value
Mean number of lifetime tweets (SD)	334.1 (959.8)	443.5 (620.9)	748.7 (899.6)	483.1 (827.7)	.31
Mean number of users they are following (SD)	266.1 (430.2)	147.0 (195.7)	293.5 (616.3)	156.3 (342.99)	.14
Mean number of followers (SD)	64.78 (64.5)	369.1 (534.6)	1341.8 (1894.93)	526.8 (1112.4)	.40

**Table 2:** Differences between small, medium, and large LHDs reported by frequency and percentage.

Variable	Small	Medium	Large	Total	*P* value
Original tweet	851 (33%)	871 (87.1%)	856 (86.6%)	2578/3000 (85.9%)	.41
Tweet truncated	231 (50.8%)	152 (15.2%)	72 (7%)	455/3000 (15.2%)	<.001
Tweet truncated from Facebook	109 (50%)	82 (38%)	27 (12%)	218/3000 (7.3%)	<.001
Retweet	147 (35.3%)	130 (31.2%)	140 (33.6%)	417/3000 (13.9%)	.54
@Reply	3 (10%)	8 (27%)	19 (63%)	30/3000 (0.01%)	.001
@username	178 (30.1%)	184 (31.1%)	230 (38.9%)	592/3000 (19.7%)	.06
Redirect to another link, websites, etc, to learn more	706 (31.9%)	708 (32.0%)	797 (36%)	2211/3000 (73.7%)	<.001
Use of personal pronouns (1st and 2nd person)	401 (36.4%)	369 (33.5%)	332 (30.1%)	1102/3000 (36.7%)	.006
Hashtags (#)	116 (23.9%)	135 (27.8%)	235 (48.4%)	486/3000 (16.2%)	<.001
Organization—Overall	438 (36.9%)	382 (32.2%)	366 (30.9%)	1186/3000 (39.5%)	.003
Organization—Information	241 (39.2%)	201 (32.7%)	173 (28.1%)	615/1186 (51.9%)	.08
Organization—Engagement	132 (31.8%)	125 (30.1%)	158(38.1%)	415/1186 (35.0%)	<.001
Organization—Action	68 (43%)	56 (35%)	34 (22%)	158/1186 (13.3%)	.02
Personal Health—Overall	509 (30.3%)	574 (34.1%)	599 (35.6%)	1682/3000 (56.0%)	<.001
Personal Health—Information	322 (32.7%)	321 (32.6%)	341 (34.7%)	984/1682 (58.5%)	.03
Personal Health—Action	187 (26.8%)	254 (36.4%)	256 (36.7%)	697/1682 (41.4%)	.03

## Discussion

### Principal Findings

This study examined how LHDs are using Twitter to communicate with their followers. Results showed that LHDs are more likely to use Twitter to convey personal-health information compared with information about the organization. Personal-health tweets were more often factually based with encouragement to take action to learn more. Organization-based tweets were generally related to events and services LHDs provided with an invitation to attend and support the events. There were some differences between large and small LHDs.

Nearly 12% of LHDs use Twitter, which is consistent with an earlier report of 13% from NACCHO [19]. This is also fairly similar to individual use of Twitter reported at 15% [15], but lower than use of Twitter within nonprofit organizations [10], large companies [26], and state health departments [3,4]. One explanation for lower use of Twitter among LHDs compared with nonprofit organizations may relate to revenue and funding streams. Nah and Saxton [27] reported that organizations that rely on donor-based funding were more likely to use social media than those funded by the government. Although LHDs appear to be using Twitter to help fulfill their public health mission, their funding, though often tenuous, is not influenced by relationships with their Twitter followers.

In the case of lower Twitter use among LHDs compared with state health departments, it is not uncommon for state health departments to employ a larger and more diverse workforce associated with broader capacity. Whereas a state health department might have the capacity to designate a staff member as a public information officer or social media specialist, this is less likely to exist among LHDs, particularly small, understaffed LHDs funded primarily by Medicaid and Medicare with mandates to provide a range of clinical services [19].

Twitter, like other social media applications, can help public health strategically establish brands, foster relationships with consumers, and promote its organizations as well as their products and services. However, LHDs are using Twitter primarily as a way to distribute personal-health information, which is inconsistent with ways many other organizations use Twitter. While some LHDs are using Twitter for organization-based purposes, this core element and function of Twitter is clearly underutilized.

LHDs’ predominant use of Twitter to share information does relate to one of the ten essential public health services to inform, educate, and empower people [28]. This may be viewed by public health practitioners as a general mandate that can be addressed through a social media application like Twitter and may explain why LHDs post more information about personal health on Twitter compared with information about their organizations. However, there is little evidence that using Twitter as a one-way communication tool to disseminate health information is effective at improving health status.

While some evidence suggests that broad dissemination of information characterized by traditional mass media campaigns can improve population health, effective campaigns require simultaneous availability of and access to programs, services, and products that facilitate change [29]. Furthermore, broad dissemination of information ignores the fact that messages should be targeted to the intended audience. In the case of Twitter, LHDs may know nothing or very little about their followers unless they engage in dialogic communication to establish relationships. To indiscriminately post information on Twitter is inefficient. In fact, this contributes to what has been described as a fractured and cluttered media environment that can be resolved only through careful planning and testing of campaign content with intended audiences [29].

It was encouraging that at least one-third of LHD tweets attempted to engage followers, foster relationships, create networks, or build communities. These results are similar to those found by Lovejoy and Saxton in their analysis of how nonprofit organizations use social media [10]. Use of personal pronouns was present over a third of the time and more common among smaller LHDs. Additionally, evidence of effort toward dialogic communication included tweets that tended to be conversational in nature and may have used personal pronouns but were not necessarily intended for the purpose of engagement. This evidence of more conversational posting indicates LHDs may be trying to create a Twitter persona that is warm and friendly, thus making it more inviting for Twitter users to follow.

Data suggesting LHDs are trying to engage with audiences has not been reported previously. While it has been reported that state health departments are using Twitter almost exclusively for one-way communication [3,4], research indicates that when an organization’s communication is more interactive, the result is a better relationship with its consumers [30]. In turn, better relationships with consumers can lead to higher levels of engagement. As reported by Neiger et al [13] in their three-phase engagement hierarchy, use of social media should culminate in high engagement characterized by online or offline audience member involvement with the organization’s programs or services either as a partner or a participant.

There were a few differences among small and large LHDs in terms of tweet composition or content. While the strength of these associations is small, the relationships are instructive. First, large LHDs appear to be more sophisticated in the technical use of Twitter as evidenced by using hashtags more frequently. Both large and small LHDs appear to struggle with developing original content. For example, small LHDs are more likely to post truncated tweets (ie, they were posted somewhere else first such as Facebook then later appeared on Twitter). Large LHDs tended to redirect followers to other sites for more information. This indicates LHDs may lack either the technical capacity or general commitment to create original content on Twitter that more effectively develops relationships and engages followers.

The content of tweets also varied among LHDs based on size of population served. Small LHDs were more likely to post about the organization and less likely to post personal-health tweets. Since small LHDs may have less organizational capacity and may be focused on a more finite set of clinical services [19], they may be less inclined to disseminate personal-health information unrelated to their services or to attempt to modify the lifestyles of their followers. Small LHDs posting about their organization indicates they may be more interested in personal relationships with their clients and becoming acquainted with and connecting with their audiences.

In their organization-engagement tweets, small LHDs were less likely to acknowledge the activities of other organizations. Since small LHDs serve less densely populated areas and are typically located in rural, more isolated locations, it is reasonable they may be less likely to acknowledge other organizations that are more physically inaccessible and removed from their own clients.

Small LHDs more often asked followers to do something for the organization. This may be due to a limited capacity of small LHDs to provide a wide range of services or it may relate to a sense of familiarity or cohesion that might be more common within rural communities that are served by smaller LHDs. A long-held belief in the delivery of mental health services is that rural communities, represented primarily by smaller LHDs in this study, are more closely knit than urban communities [31].

### Limitations

Results should be interpreted with the following limitations in mind. First, to be included in this study LHDs had to have a Twitter account with a minimum of 50 tweets. There may be more LHDs now that would qualify for such a study. In addition, a cross-sectional survey of LHD tweets was obtained using the Twitter API during July 2012. The prevalence and type of LHD Twitter use may have changed somewhat from that point to the present. Also, in distinguishing between the primary purpose and general content of tweets (ie, information, engagement or action), coder subjectivity was a limitation. However, coders compared interpretations and resolved discrepancies thereby increasing intercoder reliability. Finally, while certain associations of data were found to be statistically significant, the Cramer’s V statistic revealed weak associations.

### Conclusions

Twitter is being adopted by LHDs, but its primary use involves disseminating one-way information on personal-health topics as well as organization-related information. There is also evidence that LHDs are starting to use Twitter to engage their audiences in conversations.

Since a paucity of evidence supports the use of Twitter or other forms of social media to disseminate one-way information as a stand-alone intervention to improve health status, LHDs should transition to more dialogic communication. More specifically, LHDs should use Twitter to develop relationships with their followers (ie, individuals and organizations) to create partnerships that leverage resources and also increase participation in LHD programs and services with the intent of improving health status [13]. In using Twitter to develop relationships, LHDs should post more original content including information about their organizations. Conversely, LHDs should post fewer truncated tweets and redirect followers less often to other sites for information.

LHDs should also develop strategic implementation and communication plans that include forethought of how Twitter or other forms of social media could be integrated and used most effectively. For example, if Twitter is used to engage audiences and increase partnerships and program participation, strategies must also be in place to activate and sustain partnerships and program participation.

If strategic communication plans identify that priority audiences prefer Twitter as a communication channel, then Twitter should be used more effectively to reach the intended audience rather than being used indiscriminately. If communication plans do not suggest that members of priority audiences have access to social media applications or they are not preferred communication channels, other forms of communication will be more appropriate.

This study has helped identify initial patterns of Twitter use among LHDs. Future research should include investigations that help determine why LHDs actually use Twitter or other forms of social media. These studies could further examine perceived benefits of engagement or the relationship between engagement and partnership and participation outcomes. Related outcomes may be of particular interest to public health funding agencies that support social media research.

With the increasing popularity of social media, public health has unprecedented opportunities to communicate directly with its audiences. As public health more fully uses social media to engage these audiences and further research clarifies how this can be done most effectively, the potential of social media to aid in change efforts that improve health status will be better understood and applied.

## Abbreviations

API: Twitter Application Programming Interface
LHD: local health department
NACHHO: National Association of County and City Health Officials
